# Real-time monitoring of immediate drug response and adaptation upon repeated treatment in a microfluidic chip system

**DOI:** 10.1007/s00204-022-03272-8

**Published:** 2022-03-19

**Authors:** Anastasiia Zuieva, Suzan Can, Franziska Boelke, Stefanie Reuter, Sebastian Schattscheider, Elfi Töpfer, Anika Westphal, Ralf Mrowka, Stefan Wölfl

**Affiliations:** 1 Institute of Pharmacy and Molecular Biotechnology, Pharmaceutical Biology, Heidelberg University, Im Neuenheimer Feld 364, 69120 Heidelberg, Germany; 2 Microfluidic ChipShop GmbH, Jena, Germany, Stockholmer Str. 20, 07747 Jena, Germany; 3 Experimentelle Nephrologie, KIM III, 12 Universitätsklinikum Jena, Stockholmer Str. 20, 07747 Jena, Germany

**Keywords:** Organ-on-a-chip system, Microfluidic culture, Luciferase, Real-time monitoring

## Abstract

Microfluidic tissue culture and organ-on-a-chip models provide efficient tools for drug testing in vivo and are considered to become the basis of in vitro test systems to analyze drug response, drug interactions and toxicity to complement and reduce animal testing. A major limitation is the efficient recording of drug action. Here we present an efficient experimental setup that allows long-term cultivation of cells in a microfluidic system in combination with continuous recording of luciferase reporter gene expression. The system combines a sensitive cooled luminescence camera system in combination with a custom build miniaturized incubation chamber. The setup allows to monitor time-dependent activation, but also the end of drug response. Repeated activation and recovery as well as varying durations of drug treatment periods can be monitored, and different modes of drug activity can be visualized.

**Supplementary Information:**

The online version contains supplementary material available at 10.1007/s00204-022-03272-8.

## Introduction

The development of a new drug is a costly and time-consuming process, which can take over 12 years and only 1 of 10 applicants succeeds with the final approval (Dimasi et al. 2010; Hay et al. 2014). The reason for such low approval rates often lies in the incapacity of pre-clinical in vitro studies to represent the physiology of the human body and the low prediction quality of results. In consequence, the choice of pre-clinical testing method has a crucial impact on the final success rate.

Animal testing and conventional two-dimensional cell culture are two methods presently used in preclinical studies (Low and Tagle 2017). Due to genetic and physiological differences, the comparability of the results in animal testing to the actual outcome in the human body is problematic, apart from ethical concerns of this method (Sharma and McNeill 2009; Seok et al. 2013). Giving consideration to these challenges, the method of choice for early drug candidate screening often is a conventional two-dimensional cell culture system as a cost saving and high-throughput method. Nevertheless, 2D-cell culture is a very simplified approach to display the complex network of processes in the human body and an extrapolation of results obtained from a multi-well plate on an organism is seen as critical. Lack of cell-microenvironment interaction as well as metabolic enzymes and altered signaling pathways are often resulting in discrepancies in pharmacodynamic and pharmacokinetic (Jiang et al. 2014; Birgersdotter et al. 2005). This demonstrates a great need for new methods for early drug testing capable of making more accurate predictions about the resulting effects on a human organism.

Recently developed 3D-based culturing techniques use scaffolds and matrices to mimic physiological microenvironment of the cells (Ravi et al. 2015; Jensen and Teng 2020). 3D spheroid and organoid culturing methods provide another option to obtain an in vivo comparable phenotype (Chua et al. 2019; Rossi et al. 2018). Furthermore, technological development in microfabrication and microfluidics allowed the fabrication of small devices which can help to close the gap between static cell culture and animal models. These devices, known as organ-on-a-chip, consist of a system of chambers and channels. This allows a constant nutrient supply of cells seeded in the chambers by perfusing the chip with a cell culture medium. It has been shown, that a flow-induced shear stress is an important component for the development of physiological functions and an environment more comparable to living organs (Huh et al. 2011; Delon et al. 2019). Previous research of our group showed that hepatic cells cultivated on a chip under fluidic conditions were able to improve the synthesis of biomarkers such as albumin and urea among others in comparison to cells in a static two-dimensional cell culture (Theobald et al. 2018). Organ-on-a-chip can either represent a single-layer co-culturing system of different cell types (Shin et al. 2012), single organs such as liver, kidney, lung or heart (Esch et al. 2015) as well as combinations of multiple organs or disease models (Bhatia and Ingber 2014).

In this paper, we demonstrate a new real-time monitoring method based on capturing bioluminescence induced by drug treatment in a microfluidic chip system.

The bioluminescence signal confirmed the activation of a specific gene of interest fused with a firefly luciferase reporter gene (Thorne et al. 2010). Typically, to perform a luciferase reporter assay, it is necessary to harvest and lyse the cells at each time point, hence the procedure is time consuming and provides end-point results. Currently, available detection kits are designed to be performed in a typical well-format plate of a static 2D-based cell culture system. Our method facilitates cell cultivation under improved microfluidic conditions with a non-invasive real-time measurement of luciferase signal.

### Cell viability in microfluidic culture

**Fig. 1:**
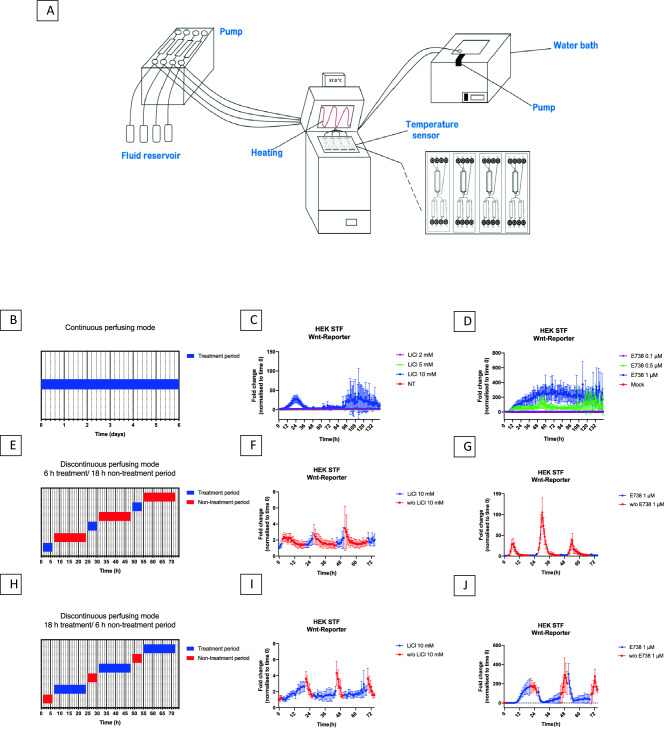
General overview and real-time luminescence measurements. **A** Schematic drawing of experimental set-up for cell cultivation on chip and direct luminescence measurement. Real-time measurements in continuous perfusing mode (**B**–**D**). **C** Treatment of HEK STF cells with 2 mM, 5 mM and 10 mM LiCl. Non-treated cells were perfused with a medium containing only D-Luciferin. **D** Treatment of HEK STF cells with 0.1 µM, 0.5 µM and 1 µM E738. Mock represents cells perfused with medium containing D-Luciferin and 0.01% DMSO. Real-time measurements in discontinuous perfusing mode (**E**–**J**). **F**, **G** Stimulation mode of HEK STF cells consisting of 6 h perfusion with 10 mM LiCl or 1 µM E738 and 18 h perfusion without treatment. **I**, **J** Stimulation mode of HEK STF cells consisting of 6 h perfusion without treatment and 18 h perfusion with 10 mM LiCl or 1 µM E738. **E**, **H** Time charts representing perfusion modes

To perform online luminescence measurement, reporter cells were cultivated in the capturing device during the treatment period. To provide a temperature of 37 °C the Biostep Celvin S^®^ capturing device was equipped with heating (Fig. 1a). For this purpose, a silicone tubing was placed in the lid of the device and connected to a pump, positioned inside a water bath and perfusing the silicone tubing with tempered water. For better heat distribution inside the capturing device, two ventilators were installed in proximity to the heating and the inner space was equipped with a sensor to monitor the temperature. For the first heating prototype, a Peltier element was used as a more compact alternative to a silicon tubing. The accumulation of the heat, created by the Peltier element, despite distribution by ventilators, resulted in cell detachment and loss. For this reason, silicone water tubing and a temperature-controlled water bath were used for heating for all further experiments. To maintain the physiological pH of the cultured cells without external CO_2_ supply, the composition of the buffering system of the cell culture medium was adjusted. Thus, the purchased minimal essential medium containing 26 mM sodium bicarbonate was supplemented with 10 mM HEPES buffer. To evaluate the comparability of the incubation conditions, one microfluidic chip was incubated in a conventional cell culture incubator at 37 °C with 5% CO_2_ in humidified atmosphere, while another chip was incubated in the Biostep Celvin S^®^ device at 37 °C. In the incubator MEM without HEPES (without phenol red, 10% FCS, 1% penicillin–streptomycin, 2 g/l glucose) was used, in the Biostep device the same medium was used but supplemented with 10 mM HEPES. Supplementary Fig. 3 shows that both conditions have the same outcome regarding cell survival. Thus, the Biostep Celvin S^®^ can be utilized for the further experimental procedure.

### Microfluidic treatment in continuous mode

Firefly luciferase signal was measured in real-time during continuous treatment of HEK293 STF reporter cells with the Wnt/ ß-catenin-pathway inductors LiCl or E738. LiCl is reported to inhibit glycogen synthase kinase-3 (GSK-3) activity, leading to accumulation of ß-catenin, binding to TCF/LEF factors and in this manner activating Wnt-pathway (Supplementary Fig. 4) (Stambolic et al. 1996). HEK293 STF reporter cells were seeded in microfluidic chips and continuously perfused for six days with either 2 mM, 5 mM or 10 mM concentrated aqueous solution of LiCl, as described in the experimental procedure. For 2 mM and 5 mM LiCl treatment, no significant fold change could be measured. An initial increase of luciferase activity could be observed during the first three hours of treatment with 10 mM LiCl, reaching the highest induction after 26 h of perfusion and decreasing to the baseline for the next three days of treatment (Fig. 1c). Another potent GSK-3 inhibitor used for the system validation is E738, a previously in our group synthetized and reported indirubin derivative (Cheng et al. 2012). Figure 1d clearly shows a concentration-dependent course of continuous treatment with 0.1 µM, 0.5 µM and 1 µM E738, resulting in a higher and longer-lasting fold change compared to LiCl treatment. An explanation for this could be that E738 leads to the accumulation of ß-catenin due to both canonical activation of the pathway as well as interaction with non-canonical signaling (Huelsken and Behrens 2002).

### Microfluidic treatment in discontinuous mode

In the next experimental setting, the impact of repeated drug treatment periods on HEK STF cells was analyzed with simultaneous luminescence measurement. Two perfusion modes were applied for this purpose. The first one with initial treatment time of 6 h followed by 18 h perfusion without drug treatment (Fig. 1e) and the second one utilizing switched periods, meaning initial 6 h perfusion without drug treatment followed by 18 h of treatment (Fig. 1h). The cycles were performed with an initial flow rate of 50 µl/h to ensure the faster exchange of medium conditions and slowed to 20 µl/h for the remaining time interval. Perfusion with 10 mM LiCl showed an increase of luminescence signal corresponding to the duration of treatment in both modes (Fig. 1f, i). In the case of 18-h treatment with 10 mM LiCl (Fig. 1i) the time span of luminescence increase was prolongedcompared to 6-h treatment. With the removal of drug treatment, the fold change started to decrease according to the progressing perfusion time without treatment. While using treatment with 1 µM E738 for 6 h the first luminescence peak appeared after withdrawal of the compound with a delay of several hours, before the signal decreased in the drug-free cultivation period (Fig. 1g). When drug exposure is maintained for 18-h with 1 µM E738 resulted the signal increased during drug exposure, but still shows a delayed response profile when compared to treatment with LiCl (Fig. 1j). In all three treatment periods with 1 mM E738 the luminescence signal remained high several hours into the drug-free period, suggesting a delayed elimination of the compound from the cells. Nevertheless, the signal is reduced upon withdrawal of the compound and induced again, upon repeated addition of the drug compound, demonstrating stable long-term cultivation of the cells and continued sensitivity in the chip system. Similar to the result of the continuous perfusion mode, E738 showed a distinct difference in the luminescence signal compared to LiCl treatment, reflecting different biological activities.

### Conclusion

In our study, we present a simple and efficient system using microfluidic on-chip tissue culture in combination with real-time recording of reporter gene expression in a non-invasive and automatized manner. Reporter gene assays based on firefly luciferase measurements are a well-established tool for the rapid analysis of gene expression and often used to analyze drug response in a tissue culture model. Usually, these types of experiments are designed in a way which requires the need of lysing the cells thereby only single end-point measurements are obtained. The experimental design presented in this paper in contrast does not require any cell lysis and therefore continuous monitoring of gene expression can be integrated into microfluidic on-chip experiments. Continuous cultivation of the cells in the microfluidic chip design also provides an improved environment for the cells compared to standard 2-dimensional cell culture more closely reflecting in vivo conditions (Wu et al. 2020; Lucchetti et al. 2021).

Moreover, reporter cell cultivation on a chip over extended time periods combined with microfluidic perfusion provides the possibility of drug treatments with different modes and time courses and the simultaneous registration of the outcome of the varying treatment protocols. Online reporter gene recording can be easily combined with different organ properties, more complex drug treatment and metabolization options (Theobald et al. 2019).

The continued microfluidic perfusion provides to adopt the cultivation to more in vivo physiologically conditions in comparison to standard static tissue culture drug activity assays in established microtiter plate cell culture assays. A further advantage of continuous online recording of reporter gene expression in on-chip microfluidic tissue cultures is the direct recording of time-dependent drug response, that can be acquired effortless due to the automatized online capturing of the signal and provides additional information on drug action (Alborzinia et al. 2011).

Thus, implementing continuous monitoring of gene expression by online recording of luciferase luminescence in a microfluidic on-chip tissue culture model will be a promising tool for pre-clinical in vitro drug research and a possible alternative to animal testing experiments. The luminesce signal generated by the STF cells is much larger than in other cell lines with other promotors (e.g. NFkB promotor, data not shown). In those cases, cameras that are more sensitive or the use of photo multipliers might be required in this setup.

## Supplementary Information

Below is the link to the electronic supplementary material.

Supplementary file1 (DOCX 3537 KB)

## References

[CR1] Alborzinia H, Can S, Holenya P, Scholl C, Lederer E, Kitanovic I (2011). Real-time monitoring of cisplatin-induced cell death. PLoS ONE.

[CR2] Bhatia SN, Ingber DE (2014). Microfluidic organs-on-chips. Nat Biotechnol [internet].

[CR3] Birgersdotter A, Sandberg R, Ernberg I (2005). Gene expression perturbation in vitro—a growing case for three-dimensional (3D) culture systems. Semin Cancer Biol.

[CR4] Cheng X, Alborzinia H, Merz KH, Steinbeisser H, Mrowka R, Scholl C (2012). Indirubin derivatives modulate TGFβ/BMP signaling at different levels and trigger ubiquitin-mediated depletion of nonactivated R-Smads. Chem Biol.

[CR5] Chua ACY, Ananthanarayanan A, Ong JJY, Wong JY, Yip A, Singh NH (2019). Hepatic spheroids used as an in vitro model to study malaria relapse. Biomaterials [internet]..

[CR6] Delon LC, Guo Z, Oszmiana A, Chien CC, Gibson R, Prestidge C (2019). A systematic investigation of the effect of the fluid shear stress on Caco-2 cells towards the optimization of epithelial organ-on-chip models. Biomaterials [internet]..

[CR7] Dimasi JA, Feldman L, Seckler A, Wilson A (2010). Trends in risks associated with new drug development: success rates for investigational drugs. Clin Pharmacol Ther [internet]..

[CR8] Esch EW, Bahinski A, Huh D (2015). Organs-on-chips at the frontiers of drug discovery. Nat Rev Drug Discov.

[CR9] Hay M, Thomas DW, Craighead JL, Economides C, Rosenthal J (2014). Clinical development success rates for investigational drugs. Nat Biotechnol.

[CR10] Huelsken J, Behrens J (2002). The Wnt signalling pathway. J Cell Sci.

[CR11] Huh D, Hamilton GA, Ingber DE (2011). From three-dimensional cell culture to organs-on-chips. Trends Cell Biol.

[CR12] Jensen C, Teng Y (2020). Is it time to start transitioning from 2D to 3D cell culture?. Front Mol Biosci.

[CR13] Jiang B, Zheng W, Zhang W, Jiang X (2014). Organs on microfluidic chips: a mini review. Sci China Chem.

[CR14] Low LA, Tagle DA (2017). Tissue chips-innovative tools for drug development and disease modeling. Lab Chip.

[CR15] Lucchetti M, Kaminska M, Oluwasegun AK, Mosig AS, Wilmes P (2021). Emulating the gut–liver axis: dissecting the microbiome’s effect on drug metabolism using multiorgan-on-chip models. Curr Opin Endocr Metab Res [internet].

[CR16] Ravi M, Paramesh V, Kaviya SR, Anuradha E, Paul Solomon FD (2015). 3D cell culture systems: advantages and applications. J Cell Physiol.

[CR17] Rossi G, Manfrin A, Lutolf MP (2018). Progress and potential in organoid research. Nat Rev Genet [internet]..

[CR18] Seok J, Warren HS, Alex GC, Michael NM, Henry VB, Xu W (2013). Genomic responses in mouse models poorly mimic human inflammatory diseases. Proc Natl Acad Sci USA.

[CR19] Sharma V, McNeill JH (2009). To scale or not to scale: the principles of dose extrapolation. Br J Pharmacol.

[CR20] Shin Y, Han S, Jeon JS, Yamamoto K, Zervantonakis IK, Sudo R, Roger D, Kamm SC (2012). Microfluidic Assay for Simultaneous Culture of Multiple Cell Types on Surfaces or within Hydrogels. Nat Protoc.

[CR21] Stambolic V, Ruel L, Woodgett JR (1996). Lithium inhibits glycogen synthase kinase-3 activity and mimics wingless signalling in intact cells. Curr Biol.

[CR22] Theobald J, Ghanem A, Wallisch P, Banaeiyan AA, Andrade-Navarro MA, Taškova K (2018). Liver-kidney-on-chip to study toxicity of drug metabolites. ACS Biomater Sci Eng.

[CR23] Theobald J, Abu el Maaty MA, Kusterer N, Wetterauer B, Wink M, Cheng X (2019). In vitro metabolic activation of vitamin D3 by using a multi-compartment microfluidic liver-kidney organ on chip platform. Sci Rep.

[CR24] Thorne N, Inglese J, Auld DS (2010). Illuminating insights into firefly luciferase and other bioluminescent reporters used in chemical biology. Chem Biol.

[CR25] Wu Q, Liu J, Wang X, Feng L, Wu J, Zhu X (2020). Organ-on-a-chip: Recent breakthroughs and future prospects. Biomed Eng Online [internet].

[CR26] Xu Q, Wang Y, Dabdoub A, Smallwood PM, Williams J, Woods C (2004). Vascular development in the retina and inner ear: Control by Norrin and Frizzled-4, a high-affinity ligand-receptor pair. Cell.

