# Supplementary material for: Real-time monitoring of immediate drug response and adaptation upon repeated treatment in a microfluidic chip system
Source: Arch Toxicol. 2022 Mar 19;96(5):1483–7. doi: 10.1007/s00204-022-03272-8 (PMC9013683; doi:10.1007/s00204-022-03272-8)
Supplement: Supplementary file 1 — Supplementary file1 (DOCX 3537 KB) [file 204_2022_3272_MOESM1_ESM.docx]

*Supplementary material*

Experimental Procedures

Test substances

LiCl (Merck, Germany) stock solution was prepared in distilled water. E738 was previously synthesized and determined by ^13^C- and ^1^H-nuclear magnetic resonance (NMR) spectroscopy ([Cheng et al., 2010](https://www.sciencedirect.com/science/article/pii/S1074552112003699?via%3Dihub#bib5)), stock solution was prepared in DMSO.

Cell line

HEK293 cells stably transfected with Super TOPFlash (STF) were kindly provided originally by Jeremy Nathans Lab (26). HEK293 STF reporter cells respond to signal transduction in canonical Wnt/ß-catenin signaling pathway and express firefly luciferase under control of seven LEF/TCF-binding sites (26).

Cell culture

HEK293 STF stable cell line was cultured in T75 flasks (Corning, Germany) in DMEM high glucose medium (GIBCO, Germany) containing 10% heat-inactivated fetal bovine serum and 1% penicillin (10000 U/ml)-streptomycin (10000 µg/ml) (GIBCO, Germany). Cells were cultured in 5% CO_2_ at 37°C in humidified atmosphere and maintained at passage number under 10.

Microfluidic cell culture

Chip design

The 3-chamber bioassay chip was manufactured by ChipShop, Jena, Germany. The chip was designed in slide format and produced of polysterol (PS-17) with dimensions of 25.5x75.5x1.5 mm. Microscopic monitoring of inserted cells was possible due to the transparency of the material. The chip consists of 1 big ant 2 small chambers, 3 inlet and four outlet ports (Supplementary figure 2a). Separate inlets and outlets allow the three chambers to be processed separately. The large chamber has a volume of 95 µl and a footprint of 47 mm^2^. The two downstream chambers are geometrically identical to each other. They each hold a volume of 27 µl and a base area of 15 mm^2^.

Static culture

The 3-chambers interaction microfluidic chips (ChipShop, Germany) were coated with 0,2 % collagen R solution (Serva, Germany) diluted 1:4 with PBS (GIBCO, Germany) via inlet 2 while blocking the inlets 1, 3 and outlets 4, 6 (Supplementary figure 2a) with male mini luer plugs (ChipShop, Germany). After the incubation time of 45 min at 37°C the diluted collagen solution was aspirated. HEK293 reporter cells were seeded using DMEM containing 10% fetal calf serum and 1% penicillin-streptomycin with a density of 4,5 x 10^4^ cells per chamber in two small chambers via outlets 4 and 6, while outlets 5, 7 and inlets 1, 2, 3 were blocked. The big chamber was filled with 200 µl PBS. After incubation over night at 37°C in 5% CO_2_ in humidified atmosphere syringe adapters were plugged into inlet 2 and outlets 5 and 7. In order to replace the old medium in the chip, a 1 ml syringe (B. Braun, Germany) without plunger was plugged into the syringe adapter of inlet 2 and filled with MEM without phenol red (GIBCO, Germany) containing 10% fetal calf serum, 1% penicillin-streptomycin, 10 mM HEPES buffer (Carl-Roth, Germany) and 2 g/l glucose (Merck, Germany). The replacement of medium took place only by gravity.

Fluidic culture

Fluidic culture was performed with neMESYS low pressure syringe pumps (Cetoni, Germany) equipped with 5 ml luer-lock plastic syringes (BD, USA). Syringes and chips were connected in two steps: first, syringes were connected with syringe adapters (ChipShop, Germany) to the silicone tubing with inner diameter 0.76 mm (ChipShop, Germany), following the connection to were normalized to area of ROI and the final result was presented as fold change of luminescence developed within first 4 hours.

Fluidic perfusion mode during treatment

For continuous drug treatment, chips were connected to polyetheretherketone (PEEK) tubing with 1/32" inner diameter. In the next step, PEEK tubing was connected through MicroTight PEEK adapter 1/32" to 1/16’’ inner diameter adapter (Fluigent, France) to PEEK capillary tubing with 360 µm inner diameter (IDEX, USA). Chips were connected to capillary tubing by male mini luer fluid connector (ChipShop, Germany). Microfluidic flow was introduced with 50 µl/h for the first 4 h after connection to the pumps to exchange the medium of all chambers, following 20 µl/h flow rate for the whole culturing period.

Real-time luciferase reporter assay

For real-time measurement in a microfluidic device, cells were perfused with MEM without phenol red medium (GIBCO, Germany) containing 10% heat-inactivated fetal bovine serum and 1% penicillin (10000 U/ml)-streptomycin (10000 µg/ml) (GIBCO, Germany), 10 mM HEPES buffer (Carl-Roth, Germany), 2 g/l glucose (Merck, Germany) and 0.1 mg/ml D-luciferin (free acid, p.j.k, Germany). Luminescence signal was measured with Biostep Celvin S^®^ (Biostep, Germany) chemiluminescence imaging system, in series of images taken every 50 minutes with integration time of 10 minutes. The signal intensity was analyzed with ImageJ Software.

The positions of reporter cell chambers were marked on positioning image using ROI manager, marked regions of interest were transferred to the luminescence images and mean intensity for each chamber was measured. Mean intensities were normalized to area of ROI and the final result was presented as fold change of luminescence developed within first 4 hours.

Fluidic perfusion mode during treatment

For continuous drug treatment, chips were connected to microfluidic pumps and perfused with medium containing the drug with following flow profile: 50 µl/h for 4 h, followed by 20 µl/h for 140 h. For discontinuous drug treatment, two perfusion modes were applied: during the first one chips were perfused with medium containing the drug with 50 µl/h for 4 h, followed by 20 µl/h for 2 h. Then syringes of the neMESYS pumps were exchanged and perfusion with medium without drug was started with an initial flow rate of 50 µl/h for 4 h, followed by 20 µl/h for 14 h. This cycle was repeated for 72 h. In the second discontinuous perfusion mode the duration time of treatment and non-treatment period was switched.

Viability assay on a chip (life-dead staining)

After each experiment a viability assay was performed to determine the number of dead cells. Chips were washed three times with 200 µL PBS and flashed with PBS solution containing 5 µM Hoechst 33342 (Sigma-Aldrich, Germany) and 4 µM Propidium iodide (PI, Cell Signaling Technology, USA). After an incubation period of 15 min at 37°C in 5% CO_2_ in humidified atmosphere, chips were washed three times with 200 µL PBS. Images were taken with fluorescence microscope BZ-9000 (Keyence, Japan) and analyzed with ImageJ. End result represents cell survival calculated from the ratio of PI-stained cells to the number of Hoechst-stained cells.


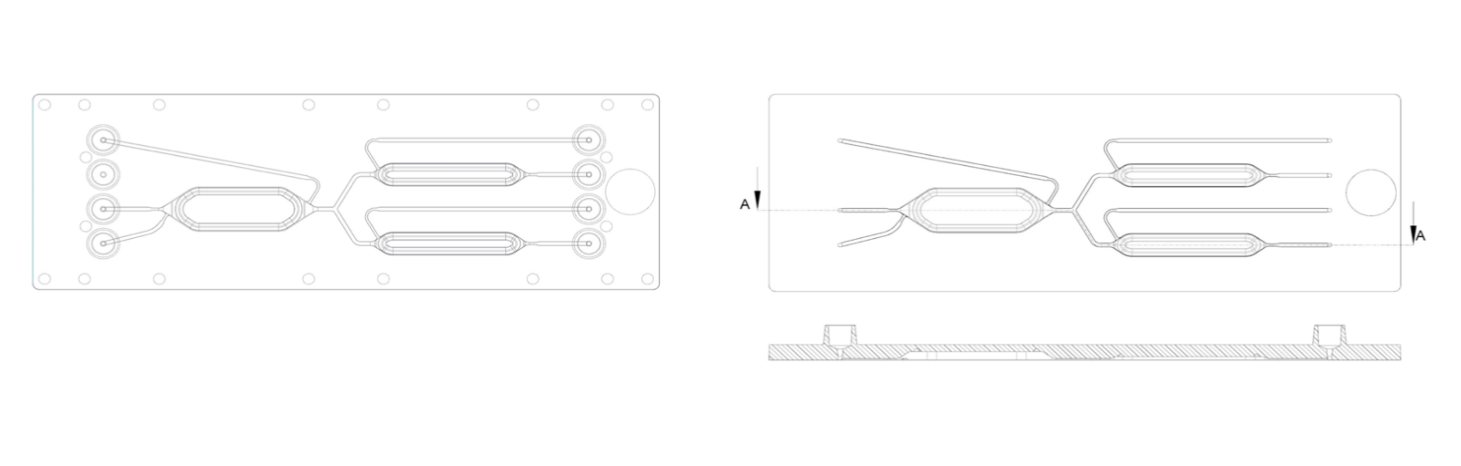


Supplementary figure 2 3-chamber bioassay chip, consisting of one big and two small chambers. A) Top view, inlets are marked with 1, 2, 3, outlets are marked with 4, 5, 6, 7. B) Section through the chip showing different volume capacity of the chambers (two small chambers are only 25% of the area of the large chamber).

A

B

1

2

3

4

6

5

7

4

4

A

C

B


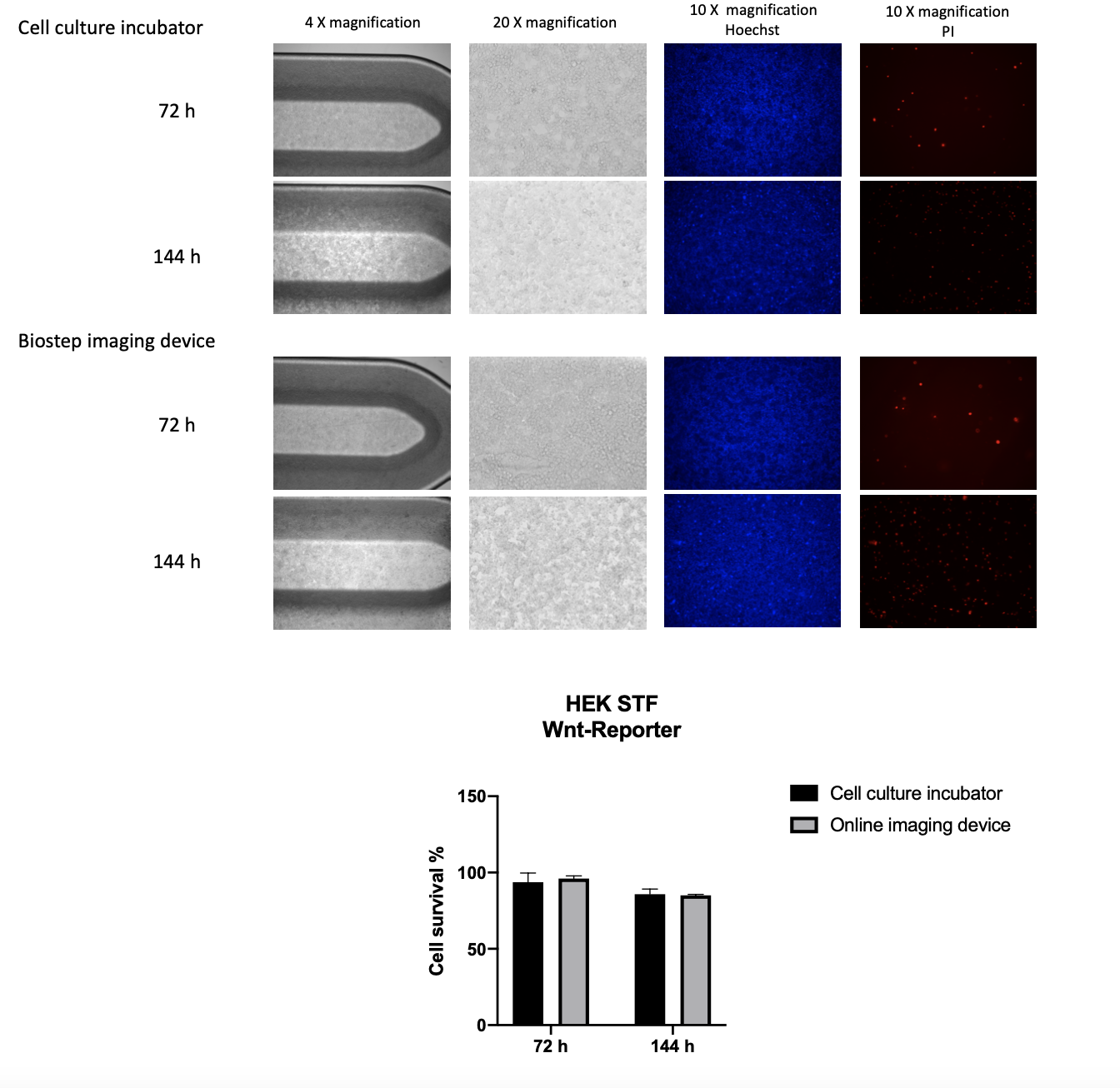


Supplementary figure 3 Evaluation of culturing conditions in capturing device vs cell culture incubator. A and B) Images were taken with a fluorescence microscope after 72 and 144 hours of cultivation either in cell culture incubator or in Biostep Celvin S^®^ device with 4 x and 20 x fold magnification (bright-field) and 10 x (blue and red channel). C) Cell survival was calculated using the count of PI-/ Hoechst-stained cells after analysis with ImageJ.


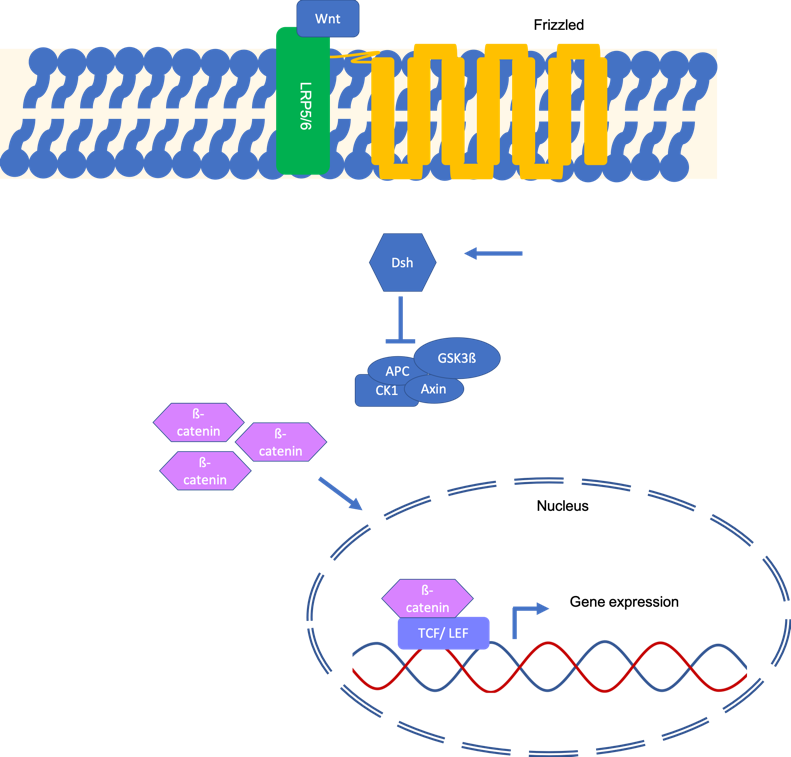


Supplementary figure 4 Canonical Wnt/ ß-catenin signaling pathway in activated state. Upon Wnt binding to frizzled transmembrane receptor and low-density lipoprotein receptor-related protein 5/6 (LRP5/6) co-receptor, dishevelled protein (Dsh) prevents ß-catenin degradation by inhibition of glycogen synthase kinase 3ß (GSK3ß). Stabilized ß-catenin translocates to the nucleus and interacts with lymphoid enhancer factor/T-cell factor (TCF/ LEF) transcriptions factor (21).

Supplementary figure 5 After each real-time luciferase measurement experiment the viability of cells on a chip was evaluated by live-dead staining. Data shows mean value with error bars representing standard deviation of at least n=3 measurements. A) and B) Live-dead staining after real-time measurements in continuous microfluidic mode. C) and D) Live-dead staining after real-time measurements in discontinuous microfluidic mode.
